# Large Emphysematous Bulla After IQOS Use: A Case-Based Literature Review

**DOI:** 10.3390/diagnostics15172267

**Published:** 2025-09-08

**Authors:** Luiza Elena Corneanu, Diana Dumitrița Alupoae, Ștefan Valentin Creangă, Andreea Nicoleta Catană, Alexandra-Diana Diaconu, Ovidiu Rusalim Petris, Laurențiu Șorodoc, Cătălina Lionte

**Affiliations:** 1 Faculty of Medicine, “Grigore T. Popa” University of Medicine and Pharmacy, 700115 Iasi, Romania; corneanuluiza@yahoo.com (L.E.C.); alexandra-diana_diaconu@email.umfiasi.ro (A.-D.D.); ovidiupetris@yahoo.com (O.R.P.);; 2 Second Internal Medicine Department, Sf. Spiridon Clinical County Emergency Hospital, 700111 Iasi, Romania; 3 Thoracic Surgery Department, Sf. Spiridon Clinical County Emergency Hospital, 700111 Iasi, Romania; creangastefan17@gmail.com; 4 Infectious Disease Department, Sf. Spiridon Clinical County Emergency Hospital, 700111 Iasi, Romania; catana.andreea87@yahoo.com

**Keywords:** IQOS, heated tobacco products, pulmonary injury, emphysematous bulla

## Abstract

**Background and Clinical Significance**: Heated tobacco products (HTPs) are a re-emerging class of tobacco products that present themselves as alternatives to conventional cigarettes with reduced risks. However, recent evidence has shown potential association with lung injury. We present a case of a pulmonary complication associated with use of IQOS, a popular HTP, contributing to the growing evidence of its risks. **Case Presentation**: A 22-year-old man presented with sharp right posterior thoracic pain, antalgic dyspnea, chills which developed suddenly in the morning, and fever. He had no past medical history. He had been a conventional smoker for 2 years (1 pack-year) but had switched to IQOS for the previous 4 years. A thoracic X-ray examination showed a big emphysematous bulla, about 84/60 mm, located in the right middle pulmonary lobe. A thoracic CT scan described a cyst of 77/84/62 mm with hydroaeric level in the right lobe and another emphysema bulla of 11 mm in the inferior right lobe. A differential diagnosis was performed, and autoimmune diseases, tuberculosis and viral infections were excluded. Alpha-1 antitrypsin level was normal. Blood culture was positive for *Pseudomonas aeruginosa*. After 4 weeks of antibiotic therapy, the infection was cured. Surgery was necessary for pleuro-pulmonary release with division of adhesions of the giant bulla. **Conclusions**: Case reports of pulmonary injury associated with IQOS use need to be published, contributing to a better understanding of the product’s toxicity and health impact.

## 1. Introduction

Cigarette smoking remains a very important risk factor for lung diseases, although multiple environmental and genetic risk factors are also involved in pathogenesis. The primary target of inhaled cigarette smoke is the airway epithelium, which functions as a barrier to inhaled harmful chemicals [1]. Cigarette smoke is an exogenous source of oxidants, and generates intracellular oxidative species that disrupt cellular processes such as aerobic respiration, leading to oxidative stress which has been related to lung diseases [1,2].

Novel tobacco substitutes, such as heated tobacco products (HTPs), have emerged as healthier alternatives to cigarettes. Rather than burning, as with cigarettes, IQOS heats tobacco to approximately 350 °C to generate aerosols that contain nicotine. The manufacturers have advertised that the number of hazardous materials in HTPs is 90% lower than that in conventional combustible cigarettes [3]; however, this conclusion is not based on independent data [1]. Volatile organic compounds, polycyclic aromatic hydrocarbons, and carbon monoxide are present in IQOS smoke [4].

Some in vitro studies have reported HTP toxicity. Leigh et al. reported that the cytotoxicity (higher levels of cytokines) of HTPs was higher than that of e-cigarettes, but lower than that of combustible cigarettes [5]. Active epithelial-to-mesenchymal transition in the airways of smokers, a process involving the transformation of epithelial cells into mesenchymal cells, is associated with small airway fibrosis, remodeling, and lung cancer development, especially in patients with chronic obstructive pulmonary disease [6]. After dual exposure to IQOS and conventional cigarettes, loss of cell viability, increased oxidative stress, and perturbed mitochondrial homeostasis have been demonstrated [7].

Few in vivo studies have been conducted. Exposure of mice to IQOS aerosols induces inflammatory immune-cell accumulation in the lungs and increases the level of proinflammatory cytokines in the bronchoalveolar fluid [8]. Chronic exposure to IQOS aerosols induces pulmonary emphysema predominantly via apoptosis- related pathways [9].

We report an unusual complication of IQOS use in a young male and the challenges of care in such a situation, and also review data in the literature regarding lung injury associated with HTP use.

## 2. Case Presentation

**Figure 1:**
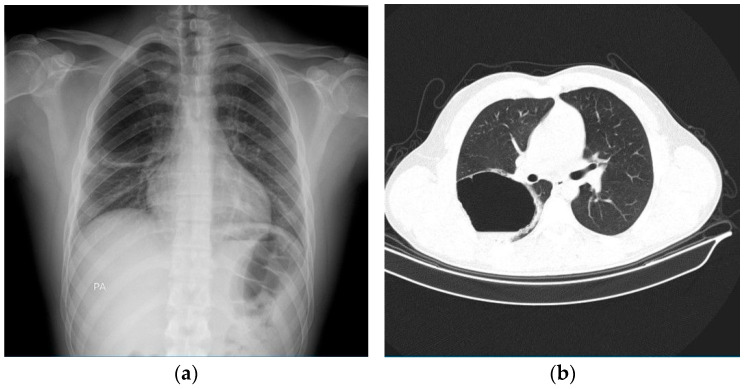
Imagistic investigations for diagnosis assessment upon admission. (**a**) Chest-X-ray shows hyper-transparent image with clear internal and external contours, homogeneous, oval, of 84/60 mm diameter (anteroposterior/craniocaudal), in the right middle lobe. (**b**) Lung CT scan shows a septate cystic lesion with air-fluid level (fluid component measuring 11 mm in thickness and air component measuring 66 mm), with a thin, irregular wall (approximately 3 mm thick), with overall dimensions of 77/84/62 mm (anteroposterior/transverse/craniocaudal), localized subpleural, in the posterior segment of the right upper lobe, with mass effect on the posterior half of the oblique fissure, apparently without communication with the bronchial tree.

A 22-year-old man presented with sharp right posterior thoracic pain which developed suddenly in the morning while sleeping, associated with shortness of breath, chills, and fever. He had no past medical history. His mother was presently suffering from bronchial asthma. He had been a smoker of combustible cigarettes for 2 years (1 pack-year), but for the past 4 years he had been smoking IQOS. Notably, a chest X-ray performed two years before presentation, as part of a pre-employment medical exam, showed no abnormalities. The physical examination recorded fever (38.4 °C), normal pulmonary auscultation, normal oxygen saturation, normal blood pressure, and tachycardia. There were no skin lesions, edema, synovitis, or joint deformities. The initial thoracic X-ray examination showed a big emphysema bulla about 84/60 mm (antero-posterior/craniocaudal), located in the right middle pulmonary lobe (Figure 1a). A thoracic CT scan with contrast was performed. This described a cystic lesion with thin walls of 77/84/62 mm (anteroposterior/transverse/craniocaudal), with hydroaeric level, localized subpleural in the posterior segment of the superior right lobe (Figure 1b), and an emphysema bulla of 11 mm in the basal medial segment of the inferior left lobe, localized subpleural. Pulmonary embolism, pneumothorax, and other bronchial and parenchymal lesions were excluded.

Blood tests showed increased inflammatory markers (C-reactive protein and neutrophilic leukocytosis) and elevated procalcitonin levels. Because the patient had only dry cough from the first week of hospitalization, a bronchoalveolar lavage was performed, with polymerase chain reaction (RT-PCR ELITE INGENIUS) testing negative for *Mycoplasma pneumoniae*, *Chlamydophila and Legionella*, and GeneXpert RIF/TB TEST for tuberculosis also negative. Bronchoscopy was deferred for technical reasons. Tests for SARS-CoV 2, influenza, and HIV infection were negative. Markers for autoimmune diseases and antibodies against *Echinococcus granulosus* were absent. Alpha-1 antitrypsin level was normal. Screening for polycystic disease was negative. During hospitalization, we performed transthoracic echocardiography, which excluded the presence of vegetations, and excluded any structural or functional abnormalities of the heart. Repeated electrocardiograms during hospital stay showed sinus tachycardia upon admission, and normal aspect thereafter.

**Figure 2:**
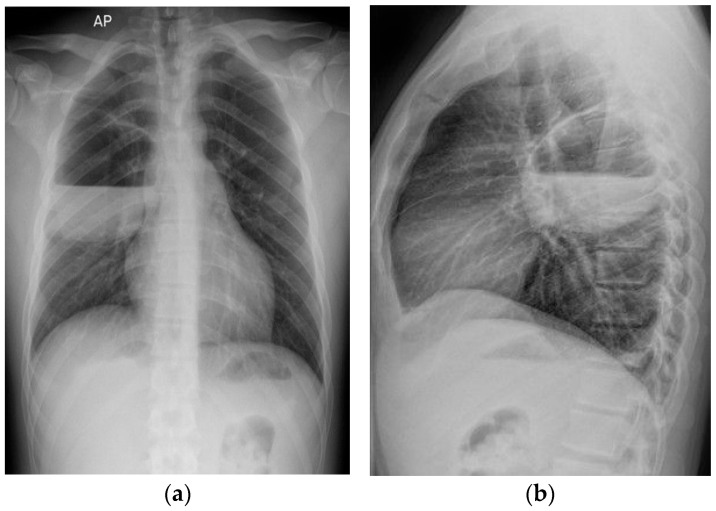
Chest-X-ray evaluation 5 days after admission shows an air-fluid image located in the right middle lobe, with 86/95/90 mm (anteroposterior/transverse/craniocaudal), well delimited with slightly irregular contour, occupied in the lower half by fluid level. (**a**) Antero-posterior (AP) chest-X-ray. (**b**) Profile-view chest X-ray.

The patient received empiric antibiotic treatment with Moxifloxacin and Vancomycin for the first three days after admission, with no improvement in symptoms, fever, or radiological aspect (Figure 2a,b).

When the blood culture returned positive for *Pseudomonas aeruginosa*, the treatment was changed according to the antibiogram, with Vancomycin, Amikacin, and Meropenem then being administered for 14 days in the hospital. The patient was then discharged and given a prescription for Levofloxacin, to be administered at home for 14 days. Smoking cessation was also recommended.

**Figure 3:**
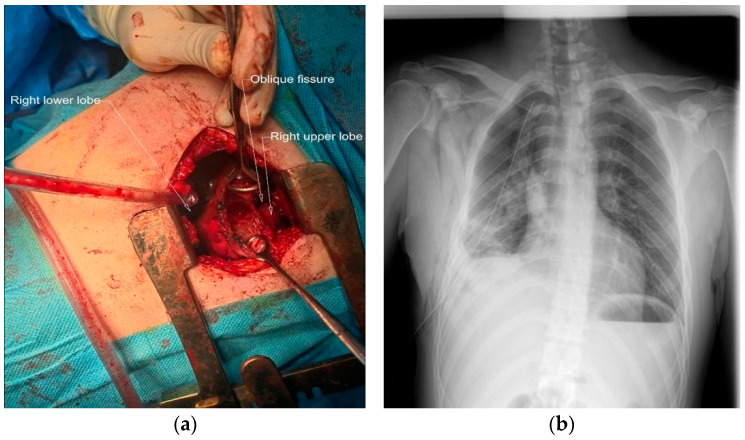
Surgical treatment of the large emphysematous bulla. (**a**) Right antero-lateral thoracotomy: incision of the emphysematous bulla in the right lower lobe and suturing of the fistula at this level. (**b**) Chest-X-ray first day post-surgery, showing a medium-intensity opacity occupying the inferior right pleural cavity.

After the resolution of the infection with antibiotic therapy, one month after discharge from the Internal Medicine Department, he was admitted to the Thoracic Surgery Department. There, video-assisted thoracoscopy surgery (VATS) was performed. However, because of the extensive pleural adhesions, a conversion was made to right anterolateral thoracotomy for pleuro-pulmonary release with division of adhesions. After the release of the lung, the bulla was incised, revealing a fistula about 3 mm wide with an air leak which was sutured with 3.0 PDO (polydioxanone) thread. The bulla was empty when the surgery was completed, without any fluid inside. There were no leaks after the suture when the lung was immersed in serum. The small bulla was then resected with the monopolar electric cautery, and the big bulla was upholstered with 2.0 Dacril threads. A large resection of the lung was not performed, in order to save as much of the lung tissue as possible. No reinforcement was needed. A single drainage was performed (Figure 3a). The use of a single drain when there are no air leaks is common.

**Figure 4:**
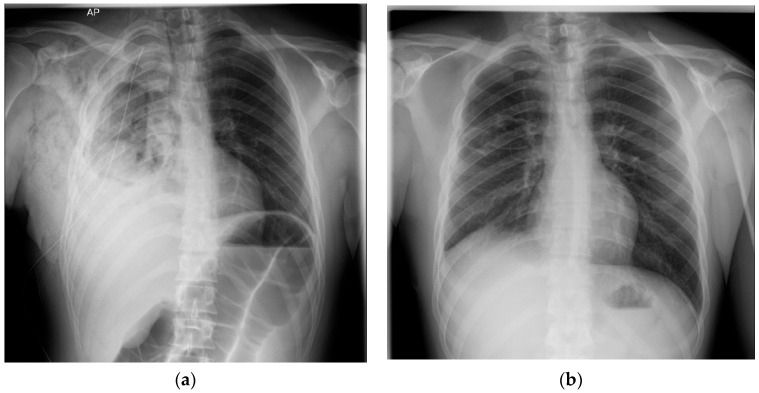
Post-surgery radiological evaluation. (**a**) Chest-X-ray the third day after surgery shows multiple heterogenous opacities occupying two-thirds of the right pleural cavity (right intrapleural hematoma), an extrapleural right hematoma, and a large bowel distension. (**b**) Chest-X-ray two-weeks after discharge from Thoracic Surgery Department showing lung re-expansion and a medium-intensity opacity occupying the right costo-diaphragmatic recess.

The chest X-rays on days 1 (Figure 3b) and 2 post-surgery showed complete lung re-expansion. The drainage was 200 mL serosanguineous/day in the first 2 days postoperative. On the third postoperative day, the patient presented with worsening chest pain, abdominal distension, and 600 mL of bloody drainage, which prompted a control chest X-ray (Figure 4a) revealing a right pleural hematoma and a colon distension. An emergency re-intervention was performed in which a right re-thoracotomy was carried out with evacuation of a 1-L pleural clot and pleural lavage with betadine solution. No active source of bleeding was found. The bleed was from the parietal pleural adhesions which were released in the first surgery, probably because of high blood pressure caused by post-operative pain. The patient needed high doses of analgesics postoperatively, including opioids; this led to ileus and colon distension, leading to increased abdominal pressure which contributed to the bleeding. The patient received thromboprophylaxis with Low-Molecular-Weight Heparin from day one post-operative. Local hemostasis at the level of diffuse pleural surfaces was performed. A single pleural drain was placed, which was removed on the fourth day post re-intervention. Patient evolution was slowly favorable in the Thoracic Surgery Department, but was complicated by a colitis of unknown etiology (manifested with diarrhea and fever) which responded to antibiotic therapy. Evaluation at two weeks post-discharge showed a favorable evolution after surgery (Figure 4b), and normal blood tests.

**Figure 5:**
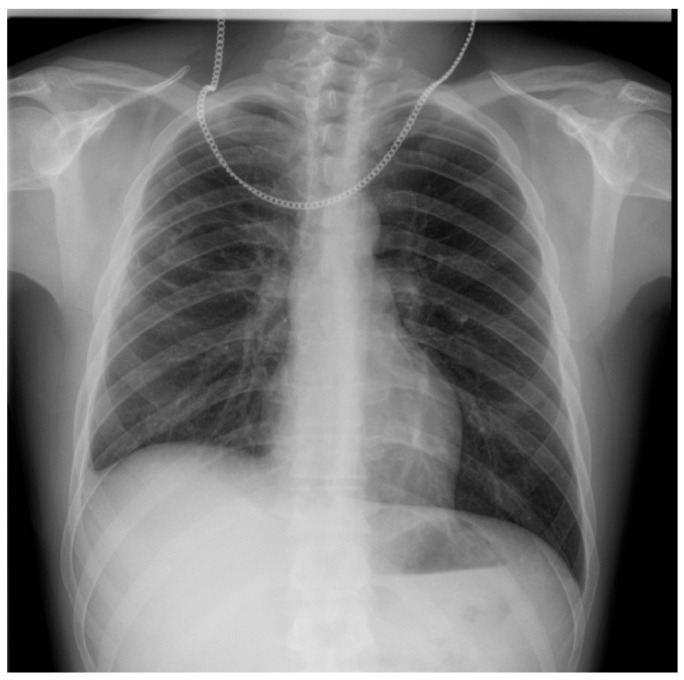
Chest-X-ray one-month after discharge from the Thoracic Surgery Department showing resolution of pleural fluid, complete lung re-expansion after surgical removal of large emphysematous bulla in the inferior right lobe.

The chest X-ray six-weeks after surgery was within normal parameters, showing no pleural collection and a complete lung re-expansion (Figure 5).

## 3. Discussion

**Figure 6:**
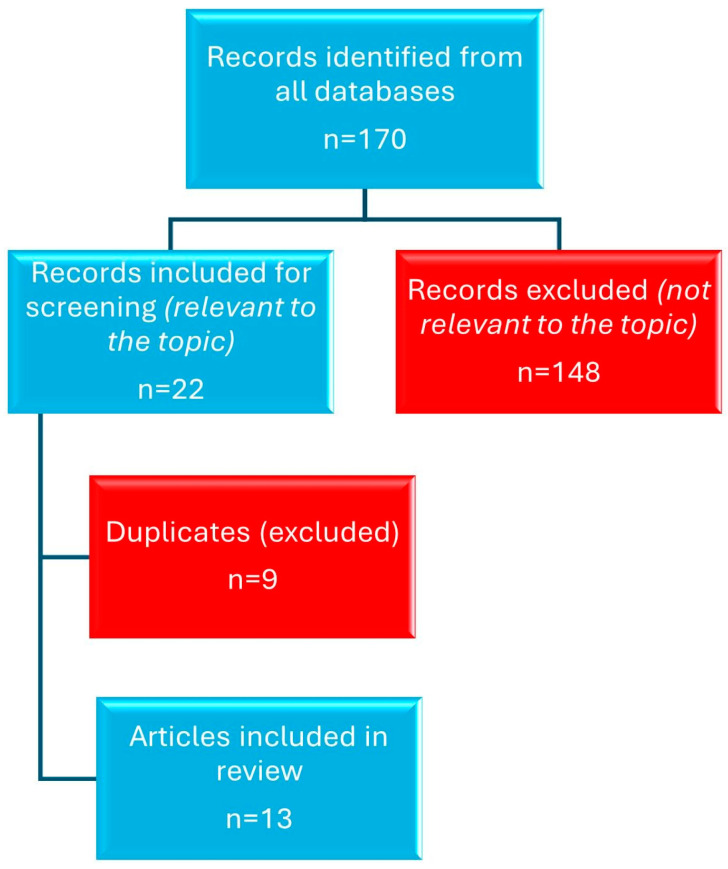
Flowchart of article selection according to CARE guidelines.

A structured literature search was conducted across multiple academic databases (Figure 6), including Clarivate, PubMed, Scopus, EMBASE, SpringerLink, Wiley, BMJ Journals, Nature, and ClinicalKey. The search strategy utilized the following keywords: *“heated tobacco products”*, *“IQOS”*, and *“lung injury”*. A total of 170 articles were initially retrieved. Following title and abstract screening, 22 articles were identified as relevant to the topic of our research, specifically addressing the pulmonary consequences of heated tobacco product use. Among these, 9 articles were duplicates found across multiple databases and were subsequently excluded. The remaining 13 articles were retained for qualitative analysis. The other 148 articles were deemed irrelevant to the clinical or pathophysiological focus of our report and were not included in the final discussion. Six clinical case reports documented acute or subacute lung injury after HTP use, while over a dozen experimental studies confirmed proinflammatory and cytotoxic effects on airway and alveolar cells.

The mainstream smoke of HTPs contains highly reactive carbonyl compounds, including acrolein and formaldehyde, at levels comparable to or only modestly lower than those in combustible cigarettes, causing cytotoxicity through oxidative damage [3]. Exposure to these compounds has been associated with acute lung injury. They contribute to epithelial damage, mucus hypersecretion, and impaired mucociliary clearance, mechanisms that could facilitate chronic infection and cyst formation [2]. However, the carbonylation of proteins by HTPs has not been sufficiently studied, and limited information is available on the prooxidant effects of HTPs on the human lung [3].

In vitro and in vivo studies consistently support a pathogenic role for IQOS aerosol in the development of lung damage. In vitro studies show that IQOS exposure in airway cells leads to oxidative stress, cell death, and impaired mitochondrial homeostasis, as well as increased levels of inflammatory cytokines (IL-8, GM-CSF), increased mucus [2], and altered immune response [7]. Moreover, products where tobacco is heated and not burned have the potential to increase microbial adherence to the airway, as with combustible cigarettes [10]. In murine models, chronic IQOS exposure resulted in peribronchiolar inflammation, alveolar space enlargement, and epithelial–mesenchymal transition, suggesting structural remodeling analogous to that seen in early-stage emphysema [9].

In this case of a young patient without any history of pulmonary disease, with normal past radiological evaluation, after we excluded other potential causes of emphysema, including chronic infections, autoimmune disorders, and genetic disorders, we related the presence of the giant emphysematous bulla located in the superior part of the inferior right lobe to chronic smoking of IQOS. An additional smaller emphysematous bulla in the inferior left lobe was described after lung CT scan. We emphasize the importance of repeated imagistic tests for an accurate diagnosis, because the description of the initial chest-X-ray, which located the bulla in the superior right lobe, was amended after lung CT scan. Finally, the correct localization of the lesion was made after thoracic surgery. There are six documented case reports in the literature describing acute pulmonary injury following exposure to HTPs, as summarized in Table 1.

Recent reports in the literature highlight an expanding spectrum of pulmonary complications associated with HTPs such as IQOS. Beyond the documented eosinophilic pneumonitis [12,13,14,15] and organizing pneumonia, cases have emerged involving cystic lesions, bullous emphysema, and even interstitial fibrosis [11]. Thomas et al. described pulmonary infiltrates in an IQOS user that resolved after product cessation, reinforcing a causal link between HTP exposure and lung injury [16]. Similarly, case reports by Tajiri and Kamada have shown findings of eosinophilic pneumonia with positive bronchoalveolar lavage in young, otherwise healthy users [14,15].

Experimental data also suggests impairment of the innate immune system. IQOS usage reduced macrophage phagocytic function and delayed bacterial clearance in animal models [9]. This observation aligns with our patient’s clinical course, where bacterial superinfection (*Pseudomonas aeruginosa*) occurred in the absence of classical immunosuppression or comorbidities.

Emphysema bullae are defined as air-filled spaces larger than 1 cm which result from damaged lung parenchyma. There are three types of bullous emphysema: type I—isolated bulla without widespread emphysema, type II—subpleural bulla, and type III—widespread bulla throughout the lung [17]. The main causes of emphysema are smoking and alpha-1 antitrypsin deficiency, an inherited autosomal dominant genetic condition affecting the lungs, liver, and sometimes the skin [18]. Less common causes of emphysema and bullae, unrelated to tobacco use, are presented in Table 2.

Diagnosis is primarily made radiologically by describing the presence of a hydroaeric level within the bullae on chest X-ray or CT scan. Management of bullous emphysema centers around smoking cessation. Surgery remains necessary in some cases, particularly when the giant bulla encompasses 30% or more of a hemithorax [16], as was the situation in our case. Also, surgery for infected emphysema bulla can significantly improve patient outcomes [19].

Infection of the emphysematous bulla with *Pseudomonas aeruginosa*, a Gram-negative bacterium, may be harmless in healthy people but can cause serious infections in immunocompromised patients [20]. This patient did not have a previous condition of immunodeficiency or autoimmune disorder, only a personal history of using HTPs like IQOS. An in vivo study revealed significantly compromised clearance of bacteria from the lungs in a group of mice exposed to HTPs or combustible cigarettes, compared to mice in a cessation group [21]. Management of giant emphysematous bullae remains surgical after the resolution of the infection [17,19].

Taken together, the abovementioned reports in the literature support the hypothesis that chronic IQOS exposure may lead to silent yet progressive lung injury, particularly in young users without baseline pulmonary disease. In our case, the absence of alternative etiologies and the presence of an extensive bullous lesion point toward a potential causal relationship. A limitation of our research is that we could not exclude the influence of conventional cigarette smoking previous to IQOS use in the genesis of the large emphysematous bulla. However, the absence of symptoms and a normal chest-X-ray two years before the current presentation might be an argument that IQOS use was responsible for this lesion. Another limitation might be the impossibility of performing bronchoscopy evaluation and lung function tests before surgery. Bronchoscopy is indicated in the evaluation of fluid-filled bullae to exclude malignancy and rule out a masked mycobacterial infection [22]. However, the authors of a study which analyzed the surgical management of infected emphysema bullae in a series of cases pointed out that, in their patients, bronchoscopy showed inconclusive findings, though four out of the seven patients analyzed had a history of COPD [19]. Although histological confirmation was not possible, the clinical and radiological context, along with the exclusion of genetic, infectious, and autoimmune causes, reinforces this interpretation.

## 4. Conclusions

We report a case of an unusual complication of IQOS use in a young male, a large emphysematous bulla complicated with Pseudomonas aeruginosa infection, which was resolved after antibiotic therapy and surgery. The case highlights an unusual complication describing an organizing pattern of lung injury possibly associated with IQOS use. Further studies are needed to elucidate the mechanisms underlying the harmful effects of HTPs and inform public health policies. This case underscores the importance of monitoring and educating individuals about the potential risks of HTPs to respiratory health.

## Figures and Tables

**Table 1:** Case reports of HTP-induced lung injury.

Study	Patient	Symptoms	Chest X-Ray	Chest CT	BAL/Tracheal Secretions	Treatment
Gulensoy et al. [11]	56-year-old male who smoked 25 pack years and quit. He had been using IQOS for 2.5 years	Sudden chest pain and shortness of breath	NA	Pleural-based atelectasis and fibroatelectatic changes in the lower lobe of the right lung, fibroatelectatic changes in the left lung and pleural thickening	NA	Partial decortication and wedge resection was performed with video-assisted thoracic surgery.
Aokage et al. [12]	16-year-old man who commenced smoking HTPs two weeks before admission	Shortness of breath that gradually worsened, with respiratory failure.	Ground-glass appearance	Mosaic ground-glass shadows on the distal sides of both lungs	Eosinophils 14.7%, neutrophils 51.7%, lymphocytes 33.6%	Methylprednisolone for 3 days and veno-venous extracorporeal membrane oxygenation for 4 days.
Kang et al. [13]	22-year-old female who started smoking HTPs 2 weeks before the onset of symptoms	Dyspnea, cough and fever	Bilateral patches of pulmonary infiltration	Bilateral multifocal patchy consolidations with multiple small nodular ground-glass opacities and interlobular septal thickening	62% eosinophils in BAL, 15% lymphocytes, 14% macrophages, 4% neutrophils	Methylprednisolone
Kamada et al. [14]	20-year-old man who started smoking HTPs 6 months previously	Fever and shortness of breath	Bilateral opacities	Bilateral infiltration, smooth interlobular septal thickening and pleural effusion	BAL with 60% eosinophils, 20% lymphocytes, 15% macrophages, 5% neutrophils	Prednisolone
Tajiri et al. [15]	47-year-old woman who smoked for 27 years and switched from conventional cigarettes to smoking HTPs 4 months before the referral	Cough and fever	Bilateral infiltrate	Bilateral patchy ground-glass opacities with interlobular septal thickening	72% eosinophils in BAL, 22% macrophages, 4% lymphocytes, 2% neutrophils	Prednisolone
Thomas et al. [16]	40-year-old man who smoked for 20 years and switched from conventional cigarettes to smoking HTPs 6 months before the referral	No systemic complaints	Ill-defined nodular shadows in the right mid and lower zones.	Multiple scattered variable-sized nodules and patchy, partially solid opacities with surrounding ground glass opacification distributed sub-pleural and along the peri-bronchovascular region. Centrilobular emphysema bilaterally in the upper lobes, minimal pleural and pericardial fluid.	Bronchoscopy: 89% macrophages, 5% neutrophils, 4% lymphocytes, and 2% eosinophils.	Smoking cessation

HTPs—heated tobacco products; BAL—bronchoalveolar lavage; NA—not available.

**Table 2:** Main causes of emphysema bullae, adapted from [18].

Smoking	E-Cigarettes Marijuana Crack Cocaine
Infections	HIV Pneumocystis carinii pneumonia COVID-19 infection
Systemic diseases	Polyangiitis with granulomatosis Sarcoidosis
Genetic disorders	Sialic acid storage or Salla disease with impaired removal of sialic acid from lysosomes, causing cognitive impairment, ataxia, nystagmus, and basal and centriacinar emphysema Marfan syndrome Ehlers–Danlos type IV
Autoimmune disorders	Urticarial vasculitis syndrome with hypocomplementemia, a combination of urticaria, arthralgia, and angioedema, associated with panacinar emphysema Sjögren’s disease

## Data Availability

The original contributions presented in this study are included in the article.

## Abbreviations

The following abbreviations are used in this manuscript:

HTPs	Heated Tobacco Products
Rx	Radiography
CT	Computer Tomography
IL	Interleukin
GM-CSF	Granulocyte-Macrophage Colony-Stimulating Factor
NA	Not Available
BAL	Bronchoalveolar lavage
VATS	Video-Assisted Thoracic Surgery

## References

[1] Ghazi S., Song M.-A., El-Hellani A. (2024). A Scoping Review of the Toxicity and Health Impact of IQOS. Tob. Induc. Dis..

[2] Munakata S., Ishimori K., Kitamura N., Ishikawa S., Takanami Y., Ito S. (2018). Oxidative Stress Responses in Human Bronchial Epithelial Cells Exposed to Cigarette Smoke and Vapor from Tobacco- and Nicotine-Containing Products. Regul. Toxicol. Pharmacol..

[3] Nishimoto-Kusunose S., Sawa M., Inaba Y., Ushiyama A., Ishii K., Hattori K., Ogasawara Y. (2021). Exposure to Aerosol Extract from Heated Tobacco Products Causes a Drastic Decrease of Glutathione and Protein Carbonylation in Human Lung Epithelial Cells. Biochem. Biophys. Res. Commun..

[4] Auer R., Concha-Lozano N., Jacot-Sadowski I., Cornuz J., Berthet A. (2017). Heat-Not-Burn Tobacco Cigarettes: Smoke by Any Other Name. JAMA Intern. Med..

[5] Leigh N.J., Tran P.L., O’Connor R.J., Goniewicz M.L. (2018). Cytotoxic Effects of Heated Tobacco Products (HTP) on Human Bronchial Epithelial Cells. Tob. Control.

[6] Kielan Darcy M., Mathew Suji E., Lu W., Sharma P., Sukhwinder Singh S. (2020). The Ill Effects of IQOS on Airway Cells: Let’s Not Get Burned All over Again. Am. J. Respir. Cell Mol. Biol..

[7] Saha P., Jain S., Mukherjee I., Panda S.R., Zeki A.A., Naidu V., Sharma P. (2023). The Effects of Dual IQOS and Cigarette Smoke Exposure on Airway Epithelial Cells: Implications for Lung Health and Respiratory Disease Pathogenesis. ERJ Open Res..

[8] Gu J., Gong D., Wang Y., Feng T., Zhang J., Hu S., Min L. (2023). Chronic Exposure to IQOS Results in Impaired Pulmonary Function and Lung Tissue Damage in Mice. Toxicol. Lett..

[9] Nitta N.A., Sato T., Komura M., Yoshikawa H., Suzuki Y., Mitsui A., Kuwasaki E., Takahashi F., Kodama Y., Seyama K. (2022). Exposure to the Heated Tobacco Product IQOS Generates Apoptosis-Mediated Pulmonary Emphysema in Murine Lungs. Am. J. Physiol.-Lung Cell. Mol. Physiol..

[10] Sohal S.S., Eapen M.S., Naidu V.G.M., Sharma P. (2019). IQOS Exposure Impairs Human Airway Cell Homeostasis: Direct Comparison with Traditional Cigarette and E-Cigarette. ERJ Open Res..

[11] Sayin Gülensoy E., Yüksel A., Ogan N., Umudum H., Akpinar E. (2021). Subacute Lung Injury Associated with Heated Tobacco Products. Düzce Tıp Fakültesi Derg..

[12] Aokage T., Tsukahara K., Fukuda Y., Tokioka F., Taniguchi A., Naito H., Nakao A. (2019). Heat-Not-Burn Cigarettes Induce Fulminant Acute Eosinophilic Pneumonia Requiring Extracorporeal Membrane Oxygenation. Respir. Med. Case Rep..

[13] Kang B.H., Lee D.H., Roh M.S., Um S.-J., Kim I. (2022). Acute Eosinophilic Pneumonia after Combined Use of Conventional and Heat-Not-Burn Cigarettes: A Case Report. Medicina.

[14] Kamada T., Yamashita Y., Tomioka H. (2016). Acute Eosinophilic Pneumonia Following Heat-Not-Burn Cigarette Smoking. Respirol. Case Rep..

[15] Tajiri T., Wada C., Ohkubo H., Takeda N., Fukumitsu K., Fukuda S., Kanemitsu Y., Uemura T., Takemura M., Maeno K. (2020). Acute Eosinophilic Pneumonia Induced by Switching from Conventional Cigarette Smoking to Heated Tobacco Product Smoking. Intern. Med..

[16] Thomas M., Hameed M., Alhafad S., Irfan Ul H. (2024). Heated Tobacco Product (IQOS) Induced Pulmonary Infiltrates. Respir. Med. Case Rep..

[17] Siddiqui N.A., Nookala V. Bullous Emphysema. https://www.ncbi.nlm.nih.gov/books/NBK537243/.

[18] Ralhan T., Padda I., Sethi Y., Karroum P., Fabian D., Hashmi R., Elmeligy M., Piccione G., Sharp R., Fulton M. (2024). Unusual Case of Bullous Emphysema with Superimposed Pneumonia. Radiol. Case Rep..

[19] Boudabous S., Bouacida I., Ben Hadj Yahia M., Zribi H., Marghli A. (2025). Surgical management of infected emphysema bulla: A case series. Int. J. Surg. Case Rep..

[20] Mendes O.R., Debasis Bagchi D., Das A., Downs B.W. (2023). The Challenge of Pulmonary Pseudomonas Aeruginosa Infection: How to Bridge Research and Clinical Pathology. Viral, Parasitic, Bacterial, and Fungal Infections.

[21] Bhat T.A., Kalathil S.G., Leigh N.J., Goniewicz M.L., Thanavala Y.M. (2024). Can Switching from Cigarettes to Heated Tobacco Products Reduce Consequences of Pulmonary Infection?. Respir. Res..

[22] Chandra D., Rose S.R., Carter R.B., Musher D.M., Hamill R.J. (2008). Fluid-containing emphysematous bullae: A spectrum of illness. Eur. Respir. J..

